# Rheumatoid arthritis and idiopathic pulmonary fibrosis: a bidirectional Mendelian randomisation study

**DOI:** 10.1136/thorax-2023-220856

**Published:** 2024-04-22

**Authors:** Olivia C Leavy, Leticia Kawano-Dourado, Iain D Stewart, Jennifer K Quint, Joshua J Solomon, Raphael Borie, Bruno Crestani, Louise V Wain, Gisli Jenkins, Philippe Dieudé, Cosetta Minelli

**Affiliations:** 1 Department of Population Health Sciences, University of Leicester, Leicester, UK; 2 NIHR Leicester Biomedical Research Centre, Leicester, UK; 3 Hcor Research Institute, Hcor, São Paulo, Brazil; 4 Pulmonary Division, Heart Institute (InCor), University of Sao Paulo, Sao Paulo, Brazil; 5 National Heart and Lung Institute, Imperial College London, London, UK; 6 National Institue of Health and Care Research, Imperial Biomedical Research Unit, Imperial College London, London, UK; 7 National Institutue of Health and Care Excellence Imperial Biomedical Research Unit, Imperial College London, London, UK; 8 Division of Pulmonary, Critical Care and Sleep Medicine, National Jewish Health, Denver, Colorado, USA; 9 Service de Pneumologie A Hôpital Bichat, APHP, Paris, France; 10 Université Paris Cité, Inserm, PHERE, Paris, France

**Keywords:** Idiopathic pulmonary fibrosis, Rheumatoid lung disease, Interstitial Fibrosis

## Abstract

**Background:**

A usual interstitial pneumonia (UIP) pattern of lung injury is a key feature of idiopathic pulmonary fibrosis (IPF) and is also observed in up to 40% of individuals with rheumatoid arthritis (RA)-associated interstitial lung disease (RA-ILD). The RA-UIP phenotype could result from either a causal relationship of RA on UIP or vice versa, or from a simple co-occurrence of RA and IPF due to shared demographic, genetic or environmental risk factors.

**Methods:**

We used two-sample bidirectional Mendelian randomisation (MR) to test the hypothesis of a causal effect of RA on UIP and of UIP on RA, using variants from genome-wide association studies (GWAS) of RA (separately for seropositive (18 019 cases and 991 604 controls) and seronegative (8515 cases and 1 015 471 controls) RA) and of IPF (4125 cases and 20 464 controls) as genetic instruments. Sensitivity analyses were conducted to assess the robustness of the results to violations of the MR assumptions.

**Findings:**

IPF showed a significant causal effect on seropositive RA, with developing IPF increasing the risk of seropositive RA (OR=1.06, 95% CI: 1.04 to 1.08, p<0.001) which was robust under all models. For the MR in the other direction, seropositive RA showed a significant protective effect on IPF (OR=0.93; 95% CI: 0.87 to 0.99; p=0.032), but the effect was not significant when sensitivity analyses were applied. This was likely because of bias due to exclusion of patients with RA from among the cases in the IPF GWAS, or possibly because our genetic instruments did not fully capture the effect of the complex human leucocyte antigen region, the strongest RA genetic risk factor.

**Interpretation:**

Our findings support the hypothesis that RA-UIP may be due to a cause–effect relationship between UIP and RA, rather than due to a coincidental occurrence of IPF in patients with RA. The significant causal effect of IPF on seropositive RA suggests that pathomechanisms involved in the development of UIP may promote RA, and this may help inform future guidelines on screening for ILD in patients with RA.

**WHAT IS ALREADY KNOWN ON THIS TOPIC**

- Interstitial lung disease (ILD) is common in rheumatoid arthritis (RA) with a usual interstitial pneumonia (UIP) pattern of lung injury, a key feature of idiopathic pulmonary fibrosis (IPF), being observed in up to 40% of individuals with RA-related ILD. The existence of causality or its direction (whether UIP is in the causal pathway of RA or the opposite) is however unknown as observational studies cannot be used to provide evidence of causal relationships.

**WHAT THIS STUDY ADDS**

- We use a method that can be used to infer causality, known as Mendelian randomisation, to test if there is a causal relationship between RA (seropositive and seronegative) and IPF in either direction, as opposed to a coincidental occurrence of IPF in individuals with RA. We identified a robust causal relationship between IPF and seropositive RA (developing IPF increases the risk of seropositive RA) and a statistically weaker protective effect of seropositive RA on IPF.

**HOW THIS STUDY MIGHT AFFECT RESEARCH, PRACTICE OR POLICY**

- Further research is needed to define the mechanisms by which UIP might promote development of RA. Given the availability of treatments for progressive pulmonary fibrosis, earlier assessment for ILD in patients with RA, particularly those at high risk of pulmonary fibrosis, should be considered.

## Introduction

Interstitial lung disease (ILD) is common in rheumatoid arthritis (RA) and is considered an extra-articular manifestation of RA that may occur at different stages of the disease and significantly influences prognosis.^1 2^ Studies in US populations with RA show cumulative incidence estimates of clinically significant ILD in RA in 5% of patients at 10 years, 6.3% at 15 years and 6.8% over 30 years of follow-up.^3^ In a study looking at over 40 million US death certificates, ILD was listed as a contributor to death in 6.8% of women and 9.8% of men.^1^ Though several histopathological/high-resolution CT (HRCT) patterns of lung damage in RA-ILD have been described, usual interstitial pneumonia (UIP) is the most frequent, seen in 40% of individuals with RA-ILD^4^ (where it is termed RA-UIP), followed by non-specific interstitial pneumonia (NSIP) which is seen in 30%.^1 2^

UIP is also the histopathological pattern of idiopathic pulmonary fibrosis (IPF), a progressive and fatal scarring disease of the lungs. RA-UIP and IPF share several clinical features such as a male sex predominance, older age at onset (around the sixth and seventh decade, respectively), indistinguishable patterns of ILD on HRCT, a poor prognosis^5^ and a similar magnitude of response to antifibrotic therapy.^6 7^

RA-UIP and IPF also share risk factors, including smoking and the *MUC5B* rs35705950 genetic variant (T risk allele), suggesting common pathogenic pathways.^8^ Indeed, the association between RA-ILD and *MUC5B* rs35705950 is restricted to the RA-UIP subtype of RA-ILD with a similar magnitude and direction to that reported in IPF.^8 9^ Of note, *MUC5B* rs35705950 was not found to contribute to the risk of RA without ILD.^8^ However, the restricted association of *MUC5B* rs35705950 with RA and a UIP pattern of injury (but not NSIP) raises the hypothesis that RA-UIP might in fact be a coincidental occurrence of IPF in individuals who also have RA, rather than UIP being a direct consequence of RA.^10^ In spite of these similarities, extrinsic risk factors such as metal and wood dust exposure and comorbidities such as gastro-oesophageal reflux disease and obstructive sleep apnoea that have been associated with IPF have not been reported in RA-ILD.^11^

Observational studies cannot provide strong evidence on causal relationships, nor the direction of causation, as they are vulnerable to confounding and reverse causation. Mendelian randomisation (MR) is a statistical approach that can infer causal relationships between two traits and the direction of causality using genetic variants as instrumental variables (IVs). MR can be considered as a ‘natural’ randomised control trial as the genetic variants an individual holds are randomly assigned at conception and do not vary during their lifetime, thus not being subject to confounding or reverse causation (online supplemental figure 1). For an MR study to be valid, three key assumptions about the IVs should hold: (1) they should be associated with the exposure (risk factor) of interest, (2) they should not be associated with confounders of the exposure and outcome relationship, and (3) they should not be associated with the outcome other than through the exposure (ie, no horizontal pleiotropy). MR results can be biased by horizontal pleiotropy, but there are methods available to detect and allow for pleiotropy.

10.1136/thorax-2023-220856.supp1 Supplementary data

We undertook a bidirectional MR analysis to test whether there is a causal relationship between RA and IPF in either direction (RA increasing the risk of IPF or IPF increasing the risk of RA), as opposed to a coincidental occurrence of IPF in individuals with RA. We used a two-sample approach where we derived causal estimates from separate studies of RA and IPF (where we consider IPF as a proxy for UIP in the absence of UIP genome-wide association studies (GWAS)). We undertook separate analyses for seropositive for rheumatoid factor (RF) and/or anticitrullinated protein/peptide antibody (ACPA) RA and seronegative.

## Methods

**Figure 1:**
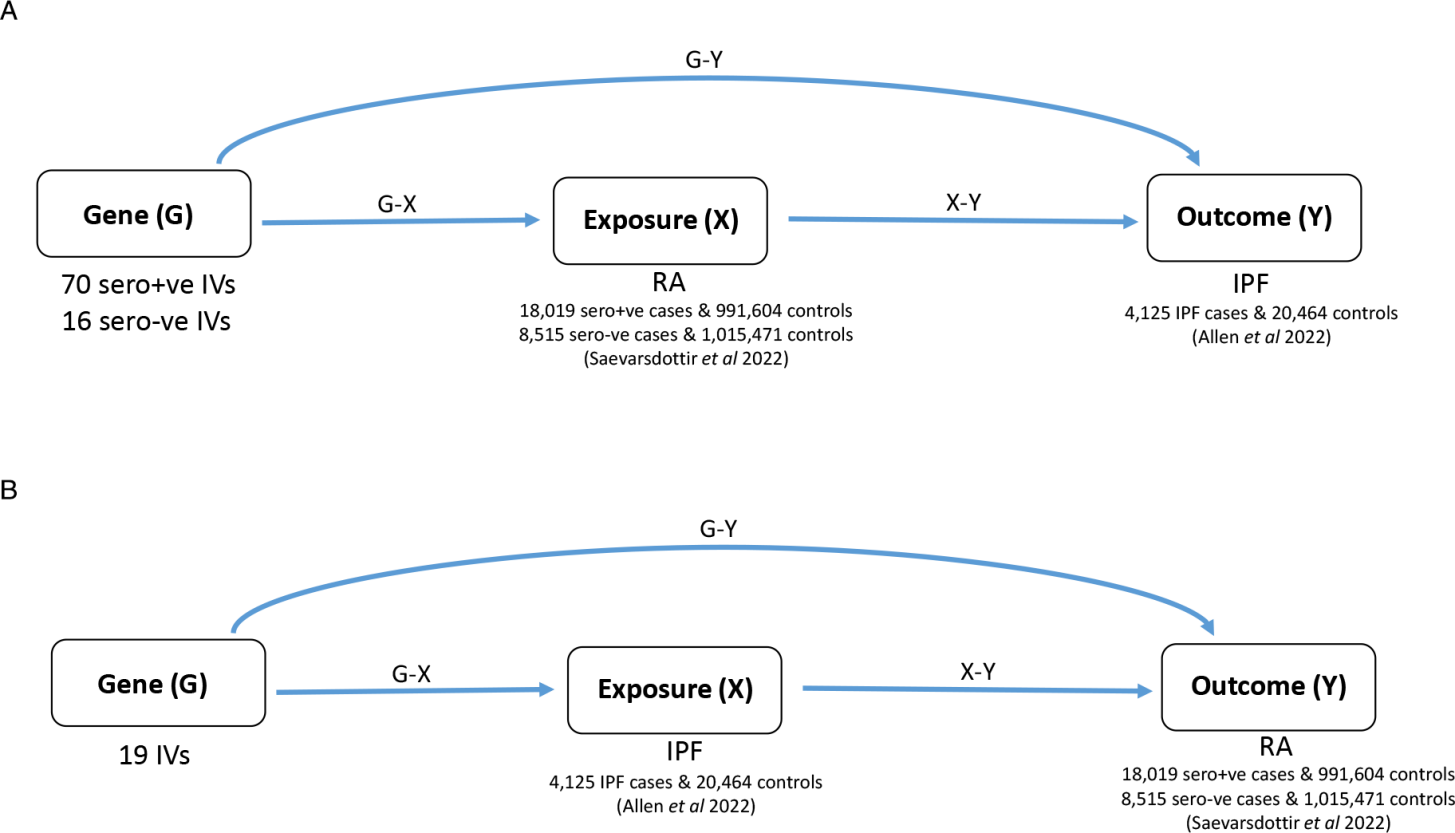
Directed acyclic graphs illustrating MR analyses and the number of instrumental variables (IVs) used for (A) RA (exposure) onto IPF (outcome) MR analysis and (B) IPF (exposure) onto RA (outcome) MR analysis. IPF, idiopathic pulmonary fibrosis; MR, Mendelian randomisation; RA, rheumatoid arthritis.

For our bidirectional MR analysis, we used a two-sample approach where summary statistics (ie, effect estimates and SEs) for the gene–exposure (‘G-X’) and gene–outcome (‘G-Y’) associations were obtained from separate studies. For the MR of the effect of RA on IPF, G-X refers to genetic associations with RA and G-Y to genetic associations with IPF, and vice versa for the MR of IPF on RA (figure 1).

### Study populations

Genetic association estimates for seropositive and seronegative RA were taken from a published study of RA^12^ that reported separate GWAS of seropositive (18 019 cases and 991 604 controls) and seronegative (8515 cases and 1 015 471 controls) RA of European ancestry (online supplemental table 1). 135 autosomal single nucleotide polymorphisms (SNPs) that were associated with RA (either seropositive, seronegative or both) in previous GWAS were selected as IVs for RA^13^ (online supplemental figure 2). These IVs were used for testing the causal effect of RA as the exposure and on IPF as the outcome.

Genetic association estimates for IPF were obtained from a previously published GWAS comprising 4125 IPF cases and 20 464 controls of European ancestry.^14^ 19 common SNPs reported as being genome-wide significantly associated with IPF were selected as IVs for IPF.^14^ These IVs were used for testing the causal effect of IPF as the exposure and on RA as the outcome.

SNPs were excluded from the analyses if they were not present in the relevant outcome dataset and no suitable proxy (linkage disequilibrium r^2^>0.8) could be found. For palindromic SNPs (ie, A/T, C/G SNPs), non-palindromic proxies (r^2^>0.8) were selected. For correlated SNPs (r^2^>0.01, determined using 1000 Genomes Project EUR population using LDlink (https://ldlink.nih.gov/), the SNP with the least significant association with the exposure was excluded.

As the use of ‘weak’ instruments can bias the results of MR, SNPs with an F-statistic <10 were excluded, where the F-statistic represents a measure of instrument strength.

### Statistical analyses

The inverse-variance weighted^15^ fixed-effect (IVW-FE) method is a fixed-effect meta-analysis of MR estimates across SNPs, where SNP-specific MR estimates are obtained using the Wald estimator (G-Y/G-X). This was used for the primary analysis for the MR in both directions, as it is the most powerful MR method in the absence of pleiotropy.

To investigate presence and magnitude of pleiotropy, the Cochran’s Q statistic and I^2^ statistic were used, respectively. Individual variant contributions to Cochran’s Q heterogeneity statistic were plotted to identify pleiotropic SNPs. In the presence of pleiotropy, a series of sensitivity analyses were conducted to account for it: inverse-variance weighted random-effect (IVW-RE) method, MR-PRESSO (MR Pleiotropy RESidual Sum and Outlier), weighted median, weighted mode-based estimation and MR-Egger. These different methods perform better in different scenarios as they make different assumptions about the nature of the underlying pleiotropy.^16^

IVW-RE is an inverse-variance weighted method where the fixed-effect meta-analysis model of IVW-FE is substituted by a random-effects model to allow for heterogeneity (as a proxy for pleiotropy). In particular, this method allows for balanced pleiotropy (random effects have a mean of zero), in the presence of which the point estimate is equivalent to the IVW-FE point estimate, but IVW-RE will have wider 95% CIs.

MR-Egger allows all SNPs to have pleiotropic effects; however, pleiotropic effects should be independent of the G-X associations. The method is affected by outliers, particularly when the G-X estimates are similar across different SNPs, which in turn can cause there to be low power to detect a causal effect. When the variation in the strength of the instruments is limited, MR-Egger is susceptible to dilution bias, which biases the MR results towards the null. The I^2^ of a meta-analysis of G-X estimates (I^2^_GX_) can be used to assess this, with lower values suggesting stronger dilution. Ideally, the I^2^_GX_ measure should be >90%; when this is not the case, MR-Egger should be performed using simulation extrapolation (SIMEX) to correct for the dilution bias.^17^

The weighted median method makes weaker assumptions about valid IVs, as it only assumes that at least half of the variants are valid instruments. This method is robust to outliers and is not as affected by the presence of a small number of pleiotropic variants as the IVW and MR-Egger methods.

The weighted mode method is also robust to outliers and it makes even weaker assumptions, only assuming that the largest (weighted) contribution of similar SNP-specific MR estimates comes from valid instruments.

MR-PRESSO can be used to identify and remove possible pleiotropic SNPs which have been detected in the MR analysis as outliers. However, the outlier test requires at least 50% of the genetic variants used as valid IVs.

We performed MR-Steiger directionality test to test the causal direction between the exposure and the outcome. We also performed a leave-one-out analysis, where each IV is excluded in turn and the analysis repeated to identify whether the results are highly influenced by a single IV, to determine whether any of the causal estimates were heavily influenced by individual instruments. All analyses were performed using packages in R (V.4.1.0), specifically *‘*MendelianRandomization*’* (for IVW-FE, IVW-RE, MR-Egger, weighted mode and weighted median), ‘MRPRESSO’ (for MR-PRESSO), *‘*simex*’* (for MR-Egger with SIMEX extension) and *‘*TwoSampleMR*’* (to harmonise the RA and IPF summary data, perform the leave-one-out analyses and perform MR-Steiger directionality test). Estimates for Cochran’s Q test and I^2^ were obtained using IVW-FE analysis.

## Results

### Selection of genetic instruments

Of the 135 IVs initially selected as IVs for RA, 2 were associated with seronegative RA only, 1 was associated with seronegative and combined RA (both seronegative and seropositive RA), 93 were associated with combined RA and 39 were associated with seropositive RA. For the seropositive analysis, we selected IVs associated with seropositive RA or combined RA and for the seronegative analysis IVs associated with seronegative RA or combined RA. In total, 70 were strong instruments (F-statistic ≥10) for seropositive RA (online supplemental table 2) and 16 were strong instruments for seronegative RA (online supplemental table 3). For IPF, all 19 association signals reported by Allen *et al* 14 were strong instruments for IPF (online supplemental tables 4 and 5).

10.1136/thorax-2023-220856.supp2 Supplementary data

### Causal estimate for seropositive RA on IPF

**Figure 2:**
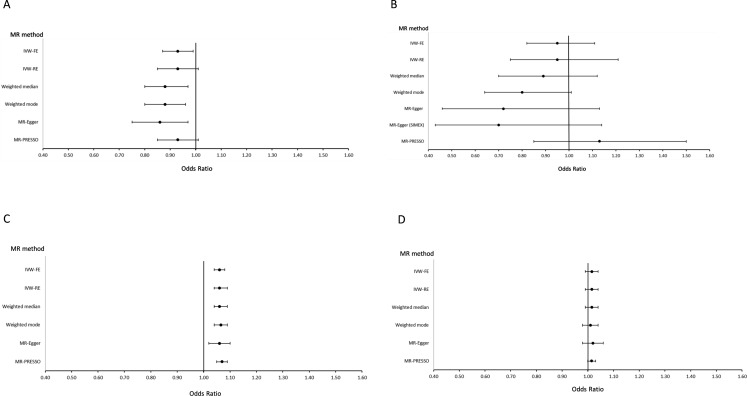
Forest plots of results of Mendelian randomisation (MR) to estimate the causal effect of (A) seropositive RA (exposure) on IPF (outcome), (B) seronegative RA (exposure) on IPF (outcome), (C) IPF (exposure) on seropositive RA (outcome) and (D) IPF (exposure) on seronegative RA (outcome). IPF, idiopathic pulmonary fibrosis; IVW-FE, inverse-variance weighted fixed effect; IVW-RE, inverse-variance weighted random effect; MR-PRESSO, MR Pleiotropy RESidual Sum and Outlier; RA, rheumatoid arthritis; SIMEX, simulation extrapolation.

The primary IVW-FE analysis gave a nominally significant result for a protective causal effect of seropositive RA on IPF (OR 0.93; 95% CI 0.87 to 0.99; p=0.032) (figure 2A and online supplemental table 6A). Although there was statistically significant evidence of pleiotropy (Q test p<0.001 and MR-PRESSO global test p<0.001), this was of moderate magnitude (I^2^=41.3%, 95% CI=22% to 56%) and no SNPs were highlighted as outliers when using MR-PRESSO or when plotting individual contributions to Cochran’s Q heterogeneity (online supplemental figure 3). Moreover, statistically significant estimates of a protective causal effect of seropositive RA on IPF were also obtained using the weighted median, weighted mode and MR-Egger analyses, and in the leave-one-out analysis, no exclusions resulted in a change in the direction of effect (online supplemental figure 4). When performing the MR-Steiger directionality test, the variance in the outcome was observed to be less than the exposure, therefore suggesting the causal direction observed is true (Steiger test p<0.05).

### Causal estimate for seronegative RA on IPF

While the IVW-FE point estimate was similar to that of seropositive RA, the CIs were very wide and the result was non-significant (95% CI 0.82 to 1.11, p=0.556) (figure 2B and online supplemental table 6A). The MR-Steiger directionality test result suggested that the causal direction observed is true (Steiger test p<0.05). The SNP rs6910071 was identified as an outlier by MR-PRESSO and there was statistically significant evidence of pleiotropy (Q test p=0.002 and MR-PRESSO global test p=0.0087, I^2^=57.8%, 95% CI=27% to 76%). Even after removing the SNP rs6910071 (positioned in the human leucocyte antigen region of chromosome 6) from the MR analysis (leave-one-out analysis), the results remained null (online supplemental figure 5).

### Causal estimate for IPF on seropositive RA

The primary IVW-FE analysis suggested that developing IPF increases the risk of seropositive RA (OR 1.06, 95% CI 1.04 to 1.08, p<0.001) (figure 2C and online supplemental table 6B). There was evidence to suggest that the causal direction observed is true (Steiger test p<0.05). There was significant evidence of pleiotropy (Q test p=0.008 and MR-PRESSO global test p=0.0313; I^2^=49.4%, 95% CI=14% to 70%). Two outliers were detected and removed by MR-PRESSO (rs112087793 and rs12912339); however, the causal effect of IPF on seropositive RA remained statistically significant (OR 1.07, 95% CI 1.05 to 1.09, p<0.001). The point estimates for the IVW-FE, weighted median, weighted mode and MR-Egger were also consistent and all results were statistically significant. In the leave-one-out analyses, no individual variant exclusions substantially attenuated the original result (online supplemental figure 6).

### Causal estimate for IPF on seronegative RA

None of the analyses suggested a statistically significant causal effect of IPF on seronegative RA (figure 2D and online supplemental table 6B), although all point estimates were greater than one, consistent with the seropositive analysis. There was no evidence of pleiotropy, MR-PRESSO did not detect any outliers and the leave-one-out analysis did not suggest that the result was influenced by any single IV (online supplemental figure 7). The MR-Steiger directionality test result suggested that the causal direction observed is true (Steiger test p<0.05).

## Discussion

This bidirectional two-sample MR study did not support the hypothesis that RA-UIP is a coincidental occurrence of IPF in patients with RA, and instead provides significant evidence of a causal effect of IPF on the development of seropositive RA (developing IPF increases the risk of seropositive RA) and a statistically weaker protective effect of RA on IPF. We note that the causal effect of IPF on RA was consistent under different models, while the protective effect of RA on IPF was more sensitive to violations of the assumptions of MR.

The rationale for a causal effect of RA on UIP has been driven by the temporal relationship between the two conditions with RA often being diagnosed before pulmonary fibrosis. However, several arguments could suggest a causal effect of IPF on RA. The loss of immune tolerance that occurs when the lungs are chronically damaged may suggest that IPF could be a risk factor for RA, as suggested by RA developing after IPF diagnosis.^18^ An independent study, the Multi-Ethnic Study of Atherosclerosis, measured RA-related autoantibodies and obtained cardiac CT scans which were assessed for subclinical ILD. This study demonstrated an association between elevated RF, ACPA and subclinical ILD, suggesting autoantibody production and pulmonary inflammation develop prior to clinical RA.^19^ However, as it was unknown if participants had RA, this may represent an association of antibodies with subclinical ILD. An assessment of patients with ILD who did not fulfil the classification criteria for RA revealed one-third of patients who were ACPA positive developed RA less than 3 years of their ILD diagnosis.^20^ Furthermore, there is a significant proportion of patients with RA (27–48%) for whom the diagnosis of ILD precedes or occurs at the same time as the onset of RA.^2 21^ Of note, a majority of individuals who developed ILD prior to RA were found to have a radiological UIP pattern.^21^ Finally, it has been shown that IgA-ACPA are elevated in up to 25% of patients with IPF and associated with changes in pathology (ie, lymphoid aggregates) also seen in established RA-UIP.^22^

The mechanism by which pulmonary fibrosis may promote RA is likely via breaching immune tolerance against citrullinated peptides. Indeed, several indirect arguments have led to the hypothesis of a mucosal origin of seropositive RA,^23^ positioning the lung as the site of initiation of the loss of tolerance against citrullinated peptides: (1) most of the environmental risk actors in seropositive RA are inhaled (smoking, silica exposure), (2) in patients with early ACPA positivity (up to 15 years before the onset of the first joint manifestations),^24^ the IgA isotype predominates,^25^ (3) the presence of peptidyl arginine deiminase 2 in lung tissue, an enzyme responsible for citrullination, and local production of ACPA,^26–28^ and (4) the existence of shared citrullinated peptide targets in lungs and joints of patients with RA.^29^ These data suggest the possibility that in a subset of patients, the citrullinated protein targets of ACPA are lung specific, leading to lung injury and fibrosis and, through a broadening of the ACPA repertoire, eventual synovitis and clinical RA.^30 31^

Mucosal inflammation has long been considered the source of ACPA associated with seropositive RA, particularly IgA isotypes, and lymphoid follicles are common in both IPF and RA.^23 28^ It is thought that chronic infection or changes in the microbiome can promote protein citrullination via chronic inflammation and NETosis.^32^ Indeed, the predominant microbiota of both IPF and RA has been found to be the phyla *Firmicutes*.^33–35^

A fundamental principle of clinical management is to treat the underlying cause of any disease. Therefore, understanding the direction of causality between two associated conditions is crucial. RA has been considered causal for UIP for many years and has guided therapeutic decisions such as prioritising the use of immunomodulatory therapy in patients with RA-UIP. Our data would suggest re-evaluating the therapeutic paradigm for treating RA-UIP. Indeed, the first randomised, double-blind, placebo-controlled trial dedicated to patients with RA-ILD identified that pirfenidone had a greater effect on slowing the decline of forced vital capacity in patients with a UIP pattern on HRCT.^6^ Second, our findings raise the intriguing hypothesis that early identification and treatment of UIP in patients with RA may offer a novel strategy for managing RA. Historically, rheumatologists have been reluctant to screen asymptomatic patients with RA for ILD at point of diagnosis and these results would suggest that this approach should be reconsidered. Lastly, our findings also suggest that pulmonologists should carefully follow the outcome of patients with IPF, paying attention to the apparition of an RA-specific autoimmunity as well as articular manifestations.

While MR provides a framework to assess causality by using genetic instruments to remove the effects of confounders and reverse causation, it does have limitations. Possible pleiotropy was detected in most of our MR analyses, although the consistency of the results across different methods allowing for pleiotropy suggests robustness of our findings. The limited number of IVs and sample size of the source GWAS that were used to derive the causal estimates for IPF and seronegative RA, compared with the seropositive RA GWAS, may have impacted power to detect a causal relationship. However, ILD is less common in seronegative RA than in seropositive RA.^36^ Our hypothesis motivating this study was that there is causal relationship between RA and UIP in either direction and so we used SNP IVs derived from IPF GWAS to model this. Having detected a causal relationship between IPF and seropositive RA, it would be relevant to understand whether the causal effect was due to mechanisms promoting the UIP pattern of lung damage. However, no GWAS specifically for UIP exists and so the IPF SNP IVs are the best proxies for UIP IVs at this time. While restricting the IPF GWAS to individuals with definite UIP may increase the strength of the instruments, the necessary HRCT or histopathology data to do this are not available in those studies. Two-sample MR assumes that the two samples are homogeneous and have the same gene–exposure association,^37^ but this may be difficult to achieve in practice when using available GWAS summary data. In our study, there are more males than females in the IPF GWAS,^14^ while females may be over-represented in the RA GWAS given that RA is more prevalent in females. Also, only the RA GWAS was adjusted for sex. A difference in the genetic effect of our instruments on the exposure by sex might introduce bias, but to our knowledge, there is no evidence to suggest that this may be the case for either RA or IPF. In addition, both the RA and IPF GWAS comprised European individuals but the RA GWAS included those from more isolated populations (Iceland and Finland) as well as other Northwestern European countries, so it might be considered a heterogeneous European population. This restriction to individuals of European ancestry also limits the generalisability of our findings to other ancestries and highlights the need for larger GWAS of IPF and RA comprising non-European populations.

It is possible that our results may be affected by biases as a consequence of the source GWAS used to define instruments. As the presence of UIP in the RA cases included in the RA source GWAS cannot be excluded, for the MR on the effect of RA on IPF, it is possible that some of the RA SNP IVs (‘G-X’) could be specifically associated with RA-UIP. These SNPs might also be associated with IPF (UIP) in which case they could introduce a bias towards a causal effect of RA on IPF; however, this was not seen. As for the MR on the effect of IPF on RA, it is also possible that RA-UIP cases in the RA source GWAS might lead to an association with the IPF SNP IVs (‘G-Y’) thereby introducing a bias towards a causal effect of IPF on RA. However, our leave-one-out analyses show that excluding the *MUC5B* SNP rs35705950, which is known to be associated with RA-UIP^8^ with an effect that is similar in magnitude to the effect on IPF, did not change the causal effect estimate (online supplemental figure 6). We also cannot exclude the possibility that the controls used for the IPF GWAS had RA, and as RA was excluded from the cases due to the specific diagnostic definition of IPF, this may have introduced some bias into the G-Y estimates; this could account for our observation of a protective effect of RA for IPF. However, this would not explain the finding of a causal effect of IPF on RA. Our MR was aimed at testing the hypothesis of a causal relationship between RA and IPF in either direction, so while our findings support a causal relationship, the magnitude of this effect should be interpreted with caution.^16^ As observational studies cannot be used to make inferences about causal relationships between traits, MR is a useful tool for testing the hypothesis that an association might have a causal link. As we have used a binary trait (IPF diagnosis as a proxy for presence of a UIP pattern of lung damage, or RA) as the exposure, we have exercised caution in our interpretation of the causal relationships; our findings suggest that the null hypothesis of no causal relationship (in either direction) should be rejected but further investigation is required to confirm the causal effect itself.^38^

In spite of these limitations, our data suggest that pathomechanisms involved in the development of UIP may promote RA. This has implications for the management of patients with RA-UIP. In addition, the causal effect of IPF increasing the risk of developing seropositive RA would provide additional support for assessment of ILD in patients with RA, especially in subgroups of patients identified as being at high risk of pulmonary fibrosis^39 40^ and for whom antifibrotic therapy might be of benefit. While we found no support for a causal effect of RA on UIP, the opposite finding of a significant protective effect of RA against development of UIP was unexpected and requires further investigation.

10.1136/thorax-2023-220856.supp3 Supplementary data

## Data Availability

Data are available upon reasonable request. IPF GWAS data are available from: https://github.com/genomicsITER/PFgenetics. RA GWAS data are available from: https://www.decode.com/summarydata/.
