# Supplementary material for: Rheumatoid arthritis and idiopathic pulmonary fibrosis: a bidirectional Mendelian randomisation study
Source: Thorax. 2024 Apr 22;79(6):538–44. doi: 10.1136/thorax-2023-220856 (PMC11137470; doi:10.1136/thorax-2023-220856)
Supplement: Supplementary data [file thorax-2023-220856supp001.pdf]

Rheumatoid arthritis and idiopathic pulmonary fibrosis: a bidirectional Mendelian randomisation study

Author names

Olivia C Leavy[1,2], Leticia Kawano-Dourado[3,4], Iain D Stewart[5], Jennifer K Quint[5], Joshua J Solomon[6], Raphael Borie[7,8], Bruno Crestani[7,8], Louise V Wain[1,2], R Gisli Jenkins[5], Philippe Dieudé[8,9], Cosetta Minelli[5]

Affiliations

[1]Department of Population Health Sciences, University of Leicester, Leicester, United Kingdom, [2]NIHR Leicester Biomedical Research Centre, The Institute for Lung Health, University of Leicester, Leicester, United Kingdom, [3]Hcor Research Institute, Hcor, Sao Paulo, Brazil, [4]Pulmonary Division, Heart Institute (InCor), University of Sao Paulo, Sao Paulo, Brazil, [5]National Heart and Lung Institute, Imperial College London, London, United Kingdom, [6]National Jewish Health, Division of Pulmonary, Critical Care and Sleep Medicine, Denver, Colorado, USA, [7]Service de Pneumologie A Hôpital Bichat, APHP, Paris, France, [8]Université Paris Cité, Inserm, PHERE, F-75018 Paris, France, [9]Service de Rhumatologie A Hôpital Bichat, APHP, Paris, France

Corresponding author

R Gisli Jenkins, National Heart and Lung Institute, Imperial College London, London, United Kingdom, E-mail: gisli.jenkins@imperial.ac.uk

Leavy OC, *et al. Thorax* 2024; 79:538–544. doi: 10.1136/thorax-2023-220856

Supplementary Tables/Figures

Supplementary Table S1: Overview of RA and IPF GWAS study populations

Phenotype	Cases	Controls	Population	PMID ID	Year
RA overall	31,313	995,377	Northwestern European:<br>Sweden (8,658 cases & 9,418 controls)<br>Denmark (7,662 cases & 86,964 controls)<br>Iceland (3,613 cases & 341,788 controls)<br>Norway (881 cases & 28,517 controls)<br>UK Biobank (5,798 cases & 402,767 controls)<br>FinnGen (4,701 cases & 125,923 controls)	PMID: 35470158	2022
Seropositive RA	18,019	991,604			
Seronegative RA	8,515	1,015,471			
IPF	4,125	20,464	European:<br>UK Study (612 cases & 3,366 controls)<br>Chicago Study (541 cases & 542 controls)<br>Colorado Study (1,515 cases & 4,683 controls)<br>UUS Study (793 cases & 9,999 controls)<br>Genentech (664 cases & 1,874 controls)	PMID: 35688625	2022
Note: RA = rheumatoid arthritis and IPF = Idiopathic pulmonary fibrosis					

Supplementary Table S2: Seropositive RA and IPF association results, F-statistics and MR causal effects for seropositive RA instrumental variables

[Excel spreadsheet: Supplementary Tables]

Supplementary Table S3: Seronegative RA and IPF association results, F-statistics and MR causal effects for seronegative RA instrumental variables

[Excel spreadsheet: Supplementary Tables]

Supplementary Table S4: IPF and seropositive RA association results, F-statistics and MR causal effects for IPF instrumental variables

[Excel spreadsheet: Supplementary Tables]

Supplementary Table S5: IPF and seronegative RA association results, F-statistics and MR causal effects for IPF instrumental variables

[Excel spreadsheet: Supplementary Tables]

Leavy OC, *et al. Thorax* 2024; 79:538–544. doi: 10.1136/thorax-2023-220856

Supplementary Table S6: Results of Mendelian Randomisation analyses to estimate the causal effect of a) seropositive RA (exposure) on IPF (outcome) and the causal effect of seronegative RA (exposure) on IPF (outcome), b) the causal effect of IPF (exposure) on seropositive RA (outcome) and the causal effect of IPF (exposure) on seronegative RA (outcome).

a) the causal effect of seropositive RA (exposure) on IPF (outcome) and the causal effect of seronegative RA (exposure) on IPF (outcome)

**Exposure/Outcome**	**No. IVs**	**Model**	**OR [95% CI]**	***P***	**Heterogeneity test**
Seropositive RA/IPF	70	IVW-FE	0.93 [0.87, 0.99]	0.032	I^2 = 41.3% [95% CI: 22%, 56%], Cochran's Q $P = 2\times10^{-4}$
		IVW-RE	0.93 [0.85, 1.01]	0.101	
		Weighted median	0.88 [0.80, 0.97]	0.010	
		Weighted mode	0.88 [0.80, 0.96]	0.005	
		MR-Egger	0.86 [0.75, 0.97]	0.017	I^2_{GX} = 97.4%
		MR-PRESSO	0.93 [0.85, 1.01]	0.105	Global test $P = 2\times10^{-4}$
Seronegative RA/IPF	16	IVW-FE	0.95 [0.82, 1.11]	0.556	I^2 = 57.7% [95% CI: 27%, 76%], Cochran's Q P = 0.002
		IVW-RE	0.95 [0.75, 1.21]	0.702	
		Weighted median	0.89 [0.70, 1.12]	0.314	
		Weighted mode	0.80 [0.64, 1.01]	0.061	
		MR-Egger	0.72 [0.46, 1.13]	0.154	I^2_{GX} = 87.6%
		MR-Egger (SIMEX)	0.70 [0.43, 1.14]	0.170	
		MR-PRESSO	1.13 [0.85, 1.50]	0.405	Global test P = 0.0087

OR = odds ratio, CI = confidence interval, MR = Mendelian Randomisation, IVW = inverse-variance weighted, FE = Fixed Effect, RE = Random Effect, SIMEX= simulation extrapolation, PRESSO = Pleiotropy RESidual Sum and Outlier

b) the causal effect of IPF (exposure) on seropositive RA (outcome) and the causal effect of IPF (exposure) on seronegative RA (outcome).

**Exposure/Outcome**	**No. IVs**	**Model**	**OR [95% CI]**	***P***	**Heterogeneity test**
IPF/Seropositive RA	19	IVW-FE	1.06 [1.04, 1.08]	1.22×10^{-11}	I^2 = 49.4% [95% CI: 14%, 70%], Cochran's Q P = 0.0080
		IVW-RE	1.06 [1.04, 1.09]	3.71×10^{-7}	
		Weighted median	1.06 [1.04, 1.09]	1.74×10^{-8}	
		Weighted mode	1.07 [1.04, 1.09]	1.02×10^{-8}	
		MR-Egger	1.06 [1.02, 1.10]	0.003	I^2_{GX} = 98.4%
		MR-PRESSO	1.07 [1.05, 1.09]	1.27×10^{-7}	Global test P = 0.0313
IPF/Seronegative RA	19	IVW-FE	1.02 [0.99, 1.04]	0.182	I^2 = 0% [95% CI: 0%, 49%], Cochran's Q P = 0.7615
		IVW-RE	1.02 [0.99, 1.04]	0.182	
		Weighted median	1.02 [0.99, 1.04]	0.296	
		Weighted mode	1.01 [0.98, 1.04]	0.408	
		MR-Egger	1.02 [0.98, 1.06]	0.296	I^2_{GX} = 98.4%
		MR-PRESSO	1.02 [1.00, 1.03]	0.140	Global test P = 0.8128

OR = odds ratio, CI = confidence interval, MR = Mendelian Randomisation, IVW = inverse-variance weighted, FE = Fixed Effect, RE = Random Effect, PRESSO = Pleiotropy RESidual Sum and Outlier

Leavy OC, *et al. Thorax* 2024; 79:538–544. doi: 10.1136/thorax-2023-220856

Supplementary Figure S1: Consort diagram to show comparison of Mendelian randomisation with randomised control trial

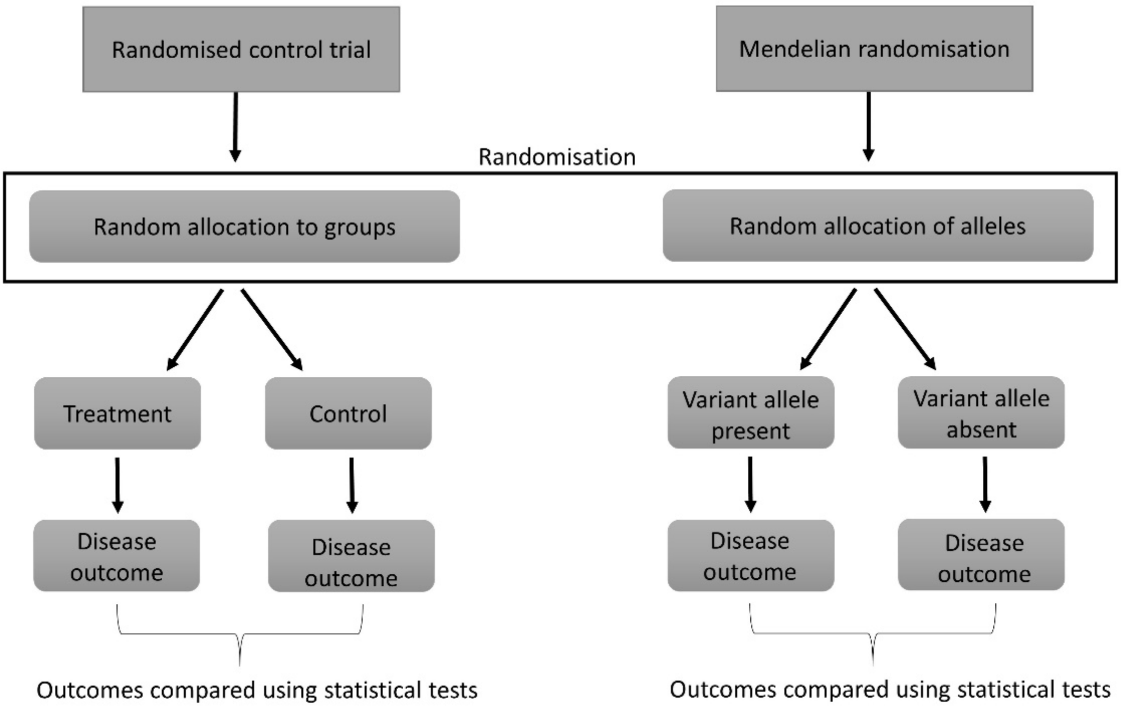

4

Supplementary Figure S2: Overview of exposure IV selection

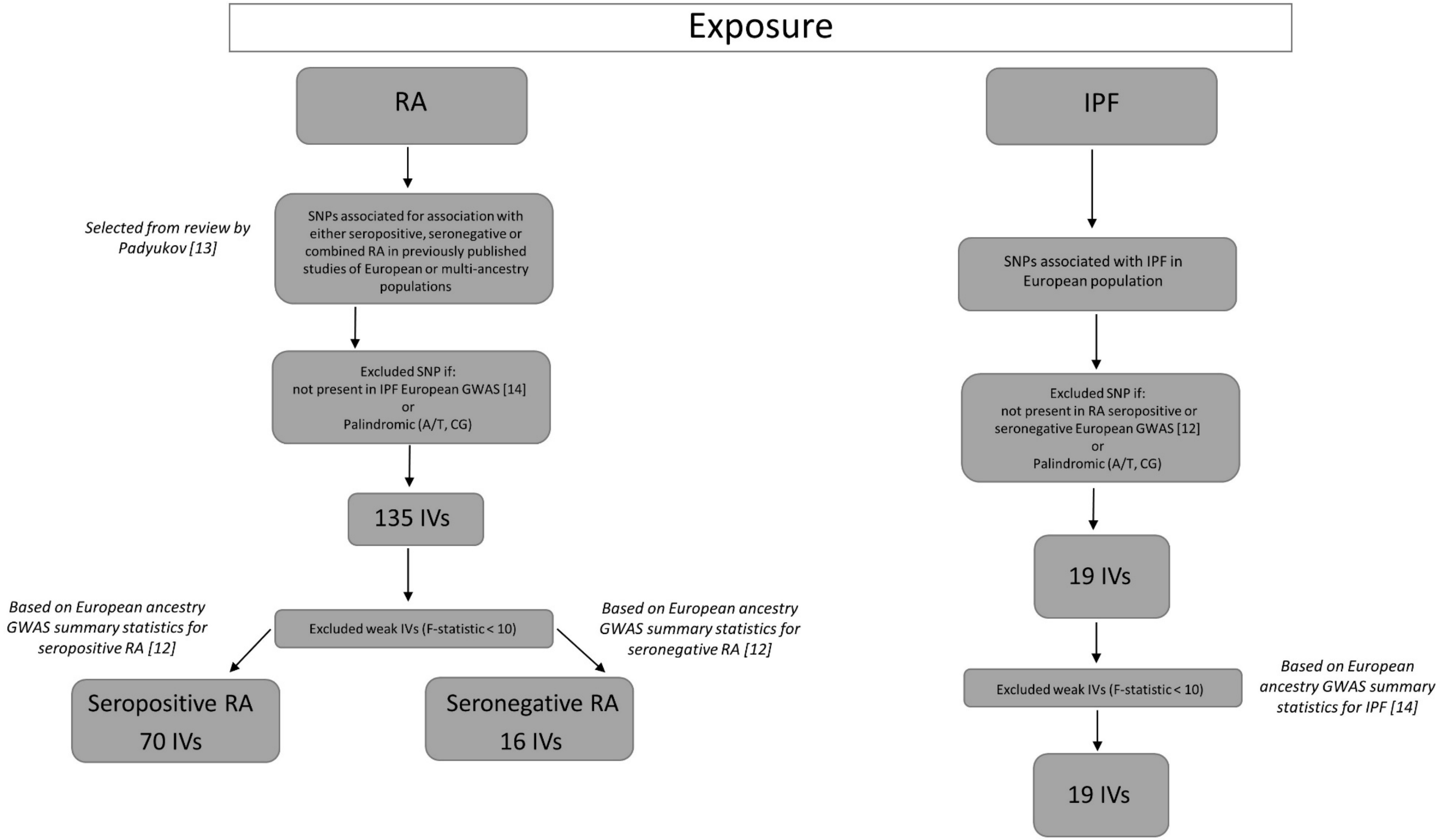

Note: RA = rheumatoid arthritis, IPF = idiopathic pulmonary fibrosis, SNP = single nucleotide polymorphism, GWAS = genome-wide association study and IVs = instrumental variables

Supplementary Figure S3: Plot of Cochran Q contribution for each seropositive RA instrumental variable

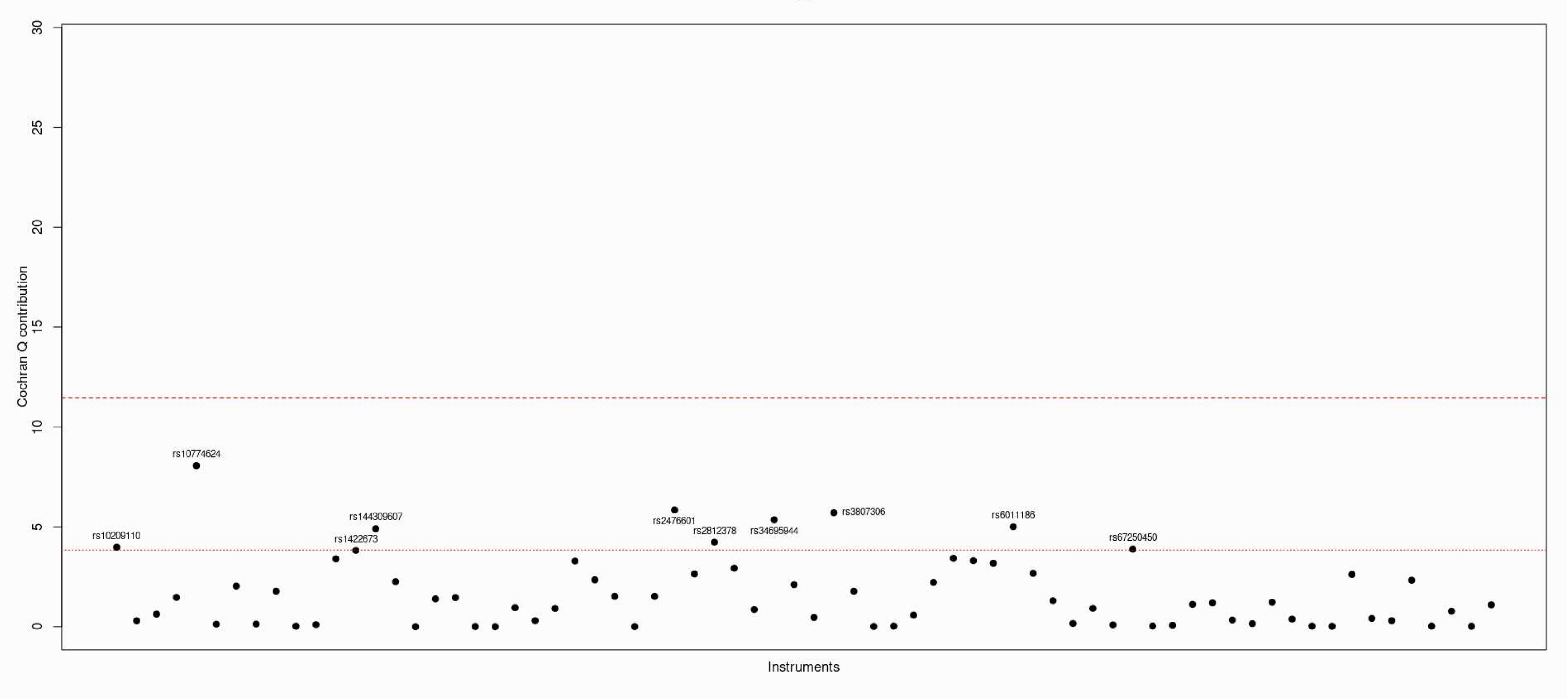

Supplementary Figure S4: Leave one out analysis for estimation of causal effect of seropositive RA on IPF

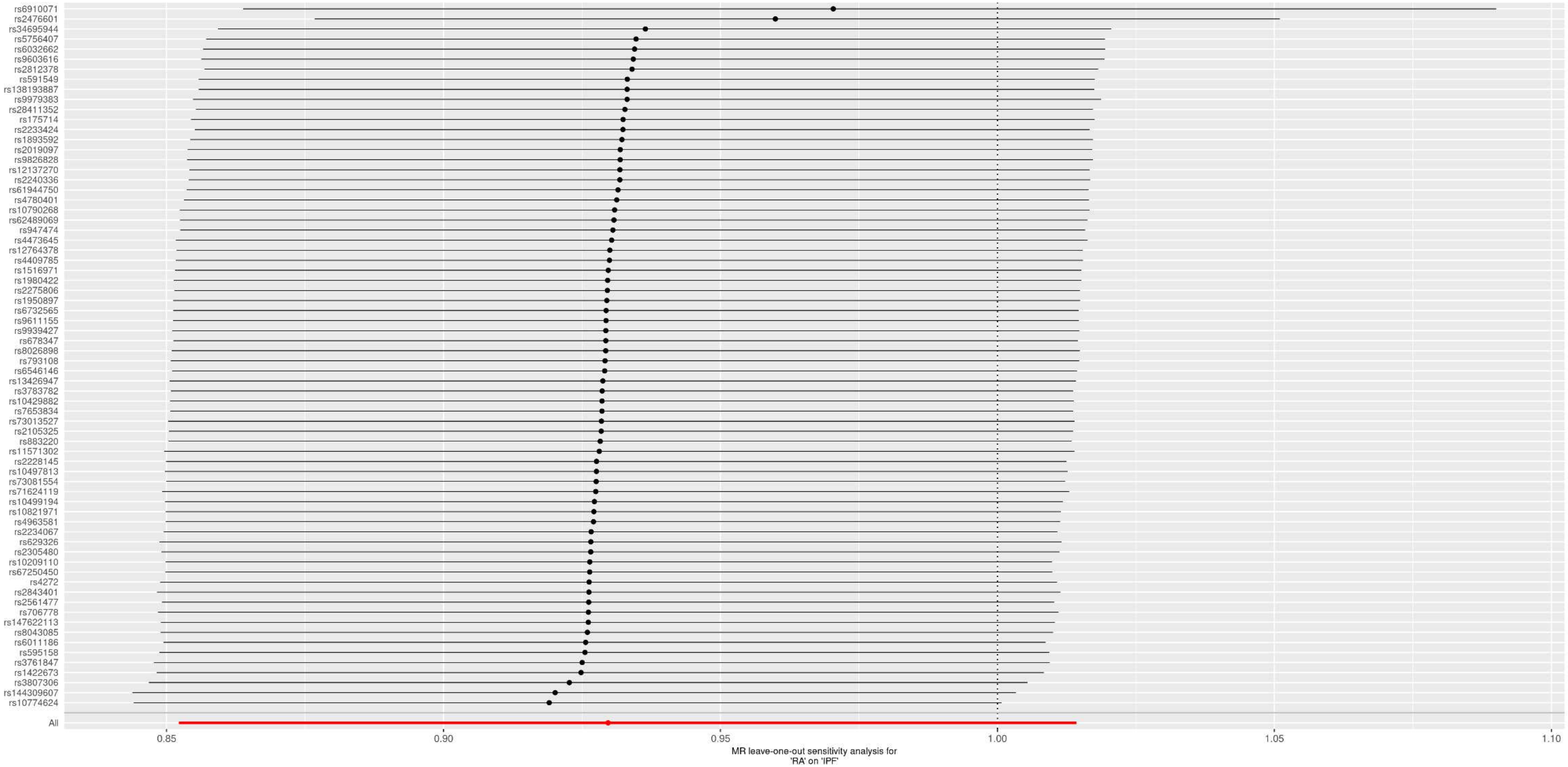

Supplementary Figure S5: Leave one out analysis for estimation of causal effect of seronegative RA on IPF

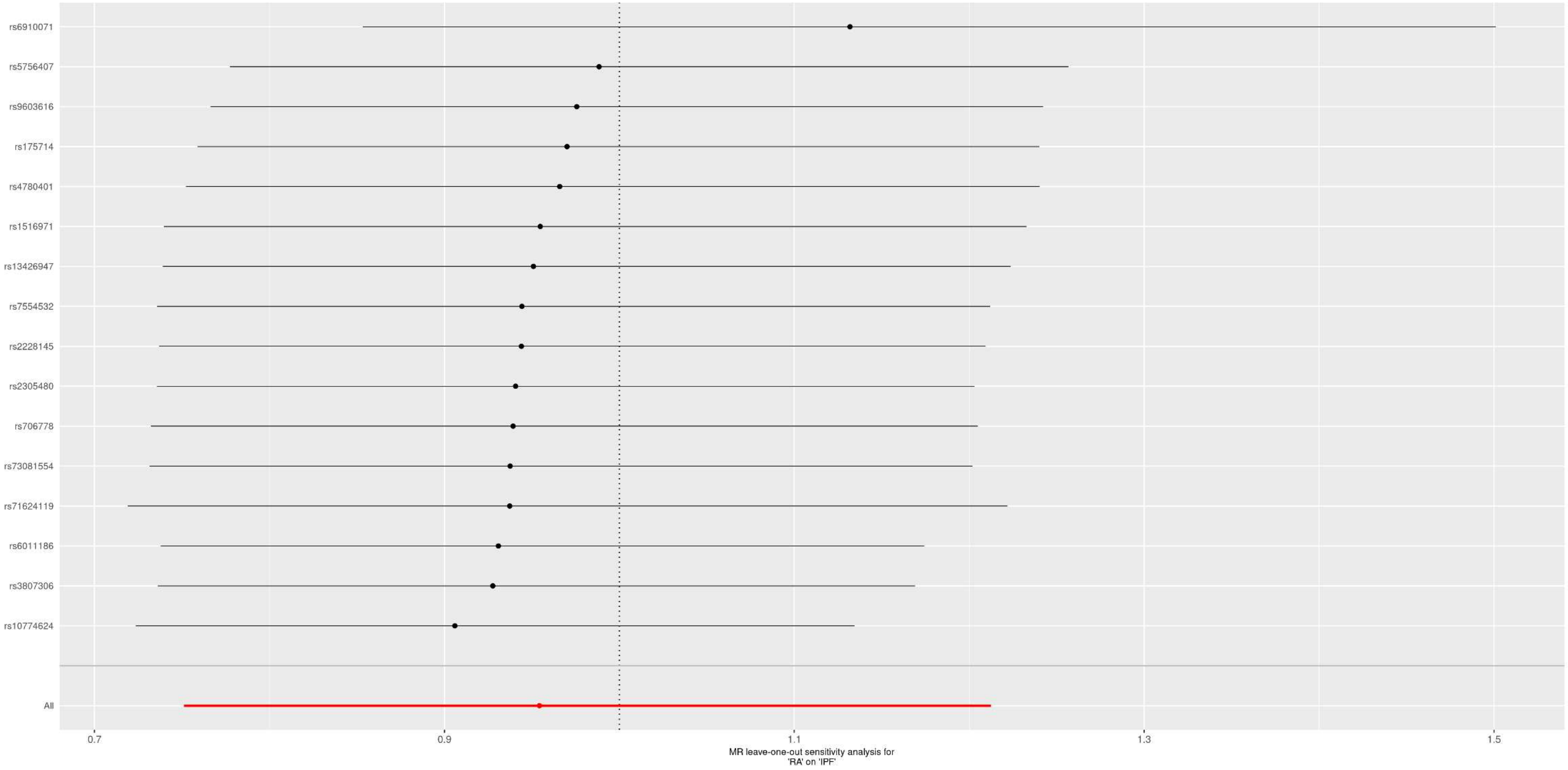

Supplementary Figure S6: Leave one out analysis for estimation of causal effect of IPF on seropositive RA

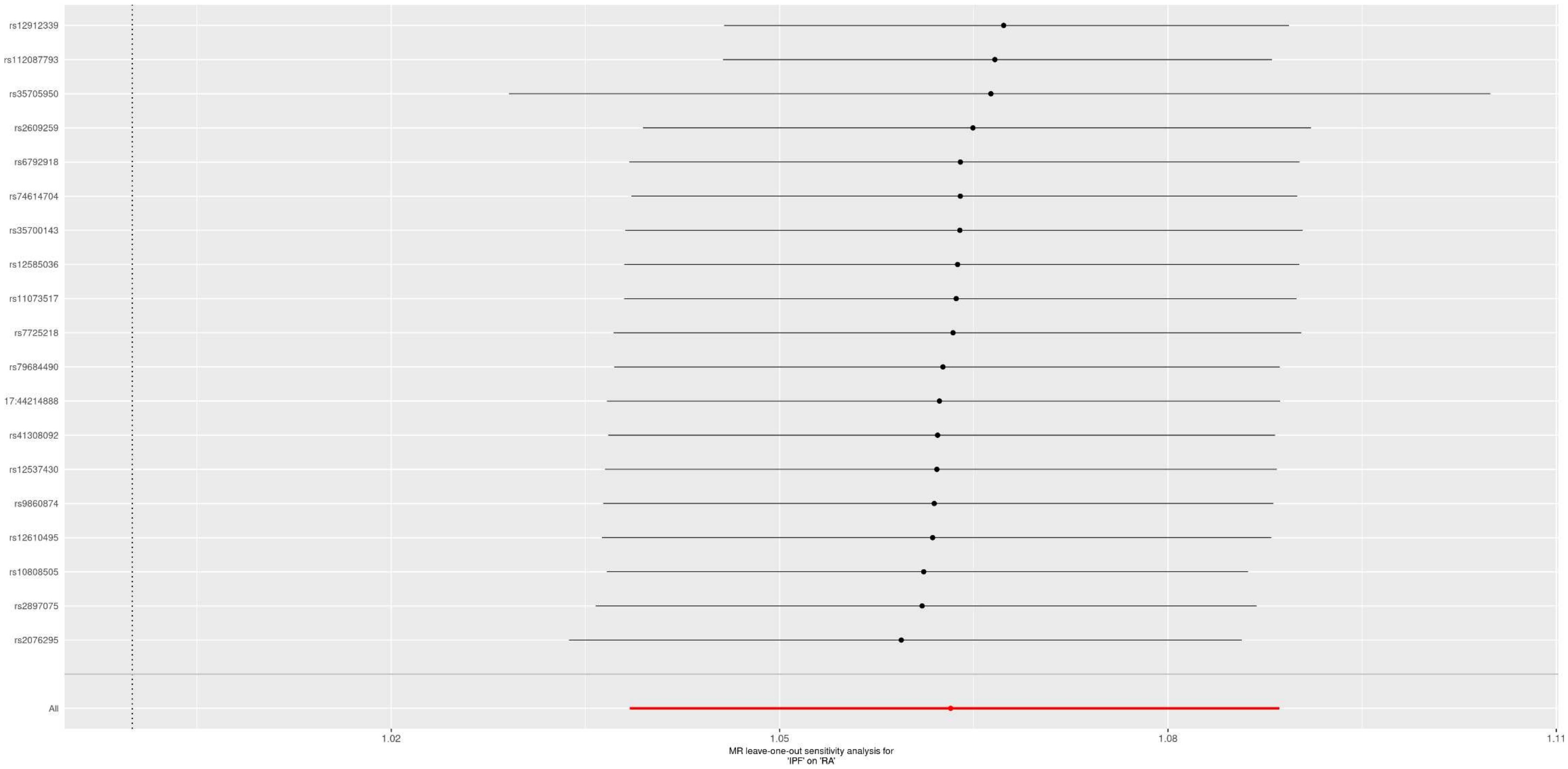

Supplementary Figure S7: Leave one out analysis for estimation of causal effect of IPF on seronegative RA

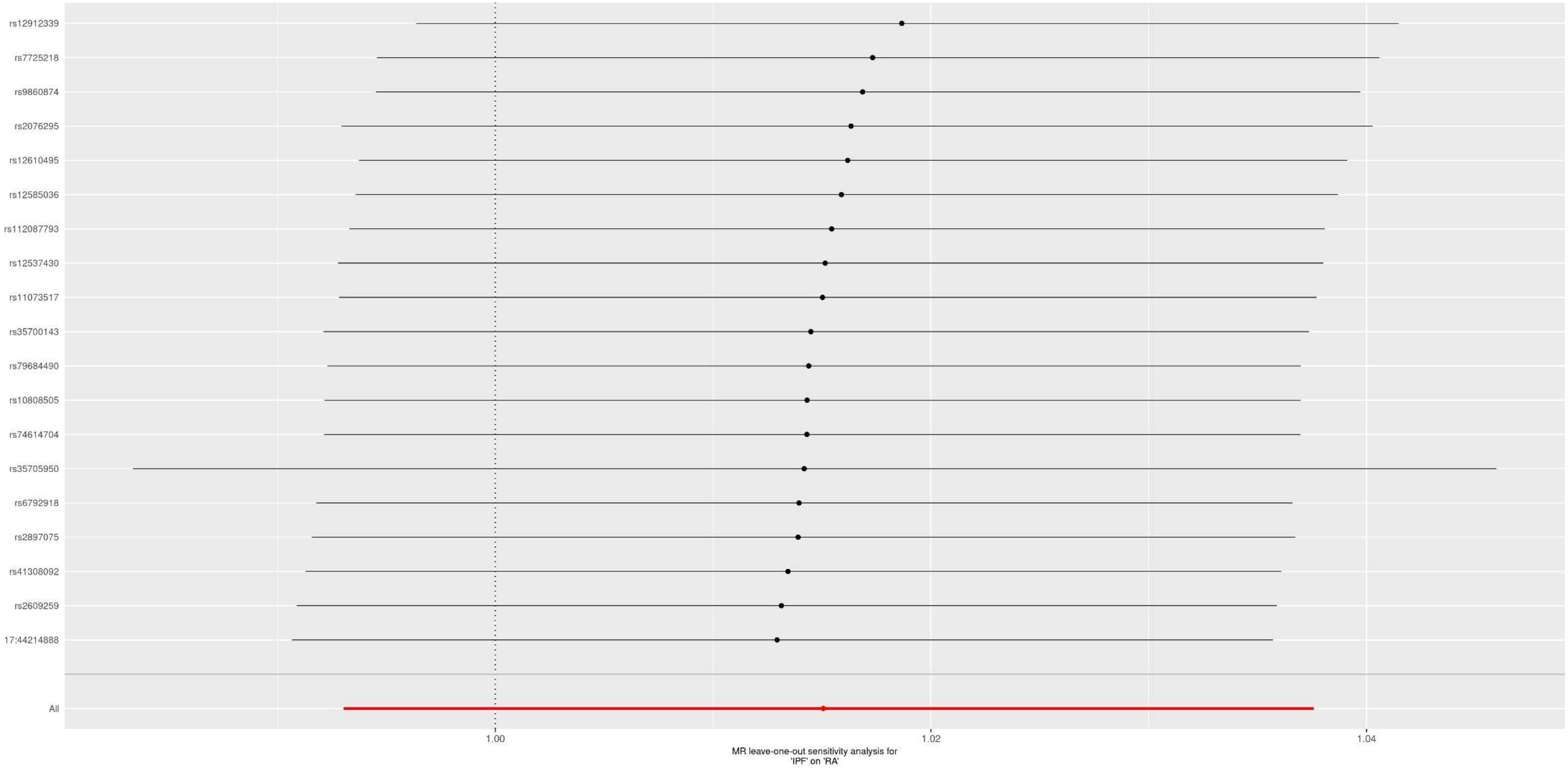

Leavy OC, *et al. Thorax* 2024; 79:538–544. doi: 10.1136/thorax-2023-220856
