# Supplementary material for: Rheumatoid arthritis and idiopathic pulmonary fibrosis: a bidirectional Mendelian randomisation study
Source: Thorax. 2024 Apr 22;79(6):538–44. doi: 10.1136/thorax-2023-220856 (PMC11137470; doi:10.1136/thorax-2023-220856)
Supplement: Supplementary data [file thorax-2023-220856supp003.pdf]

Rheumatoid arthritis and idiopathic pulmonary fibrosis: a bidirectional Mendelian randomisation study

Leavy OC, et al. *Thorax* 2024. DOI: 10.1136/thorax-2024-220856

In some patients, is RA-ILD simply a co-occurence of IPF and RA? Or is there a causal relationship?

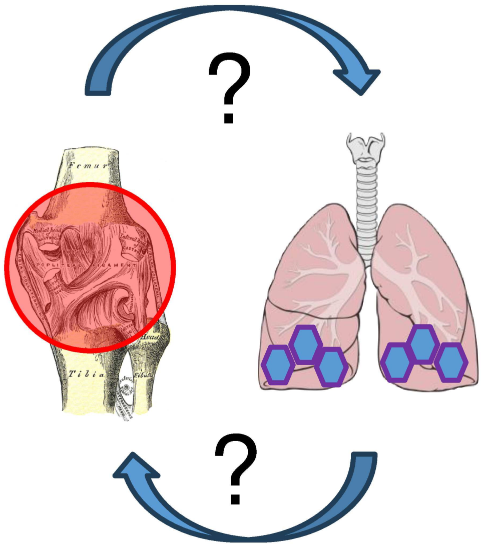

For a Mendelian Randomisation analysis, variants associated with RA (red) and IPF (purple) were used as instrumental variables (IVs).

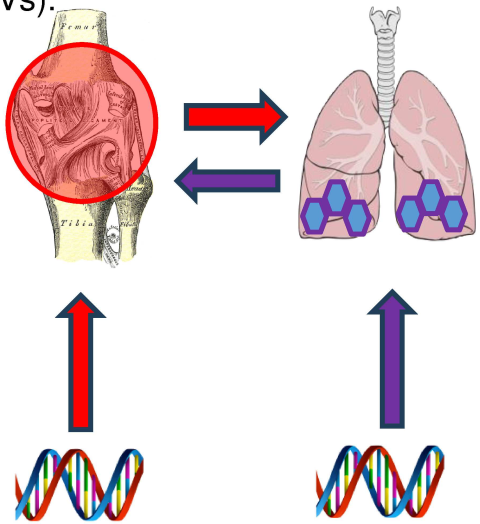

IVs for IPF were associated with increased risk of RA but not vice versa. *The causal relationship is thus likely from IPF to RA.*

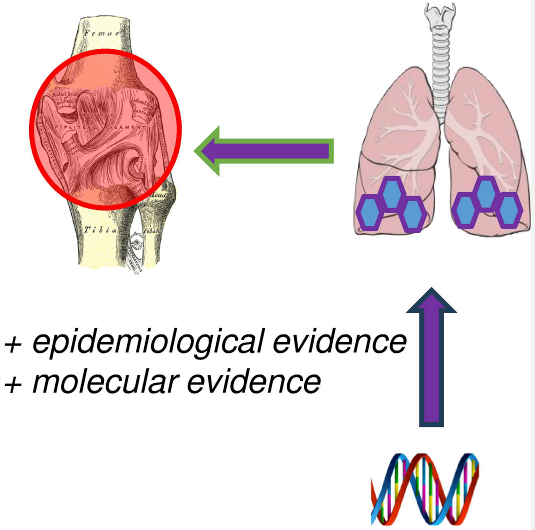

Thorax thorax.bmj.com @ThoraxBMJ British Thoracic Society BMJ
